# Sex Disparities Among Lithuanian Ischemic Stroke Patients According to Laboratory Findings; Comorbidities, Including COVID-19; Acute In-Hospital Complications; and Outcomes

**DOI:** 10.3390/medicina61081367

**Published:** 2025-07-28

**Authors:** Erika Jasukaitienė, Šarūnas Augustis, Lolita Šileikienė, Abdonas Tamošiūnas, Dalia Lukšienė, Gintarė Šakalytė, Diana Žaliaduonytė, Karolina Marcinkevičienė, Daina Krančiukaitė-Butylkinienė, Ričardas Radišauskas

**Affiliations:** 1 Department of Population Studies, Institute of Cardiology, Medical Academy, Lithuanian University of Health Sciences, LT-50162 Kaunas, Lithuania; erika.jasukaitiene@lsmu.lt (E.J.); sarunas.augustis@lsmu.lt (Š.A.); lolita.sileikiene@lsmu.lt (L.Š.); abdonas.tamosiunas@lsmu.lt (A.T.); dalia.luksiene@lsmu.lt (D.L.); karolina.marcinkeviciene@lsmu.lt (K.M.); daina.butylkiniene@lsmu.lt (D.K.-B.); 2 Institute of Cardiology, Medical Academy, Lithuanian University of Health Sciences, LT-50162 Kaunas, Lithuania; gintare.sakalyte@lsmu.lt; 3 Department of Cardiology, Medical Academy, Lithuanian University of Health Sciences, LT-50161 Kaunas, Lithuania; diana.zaliaduonyte@lsmu.lt

**Keywords:** sex disparities, ischemic stroke, COVID-19, in-hospital complications, risk, lethality

## Abstract

*Background and Objectives*: Ischemic stroke (IS) is a critical health issue, affecting individuals of all ages, sexes, and backgrounds. Mounting evidence suggests that sex indeed could play some distinct role in shaping the incidence, outcomes, and treatment of IS. In the context of the COVID-19 pandemic, contradictory findings from previous studies that also addressed sex differences in cerebrovascular diseases demonstrate the need for further focused research. This study aimed to evaluate the sex discrepancies in the clinical presentation of IS and its outcomes in patients admitted to Kaunas Hospital of the Lithuanian University of Health Sciences (LUHS), Lithuania. *Materials and Methods*: This is a retrospective record-based single-center study. All the study patients—727 men and 1082 women—enrolled between 1 January 2020, and 27 February 2022; suffered from acute IS; and had absolute contraindications against interventional IS treatment. These patients received a conservative non-interventional IS treatment at the neurological department of the LUHS’s Kaunas Hospital. The sociodemographic data; laboratory findings; comorbidities, including COVID-19; in-hospital complications; and outcome factors were obtained from the patients’ medical records and evaluated by deploying appropriate statistical tests. Hazard ratios (HRs) and 95% confidence intervals (CIs) were estimated by the Cox proportional hazards regression for in-hospital lethality. *Results*: The mean age of IS patients was significantly higher in women compared to men (*p* < 0.001), as was the proportion of in-hospital deaths (19.10% and 15.36%, respectively; *p* < 0.05). The mean total number of in-hospital complications was again significantly higher in the group of women compared to men (*p* < 0.05). The prevalence of COVID-19 was higher in men compared to women (*p* < 0.05). COVID-19 diagnosis (HR = 1.53; *p* = 0.02) and acute in-hospital pulmonary complications (HR = 1.91; *p* = 0.008) significantly increased the risk of in-hospital lethality in men. The risk of in-hospital lethality was significantly higher in women with comorbid diabetes mellitus type 2 (DM) compared to those with comorbid isolated arterial hypertension (AH) (HR = 2.25, *p* = 0.007). Increased C-reactive protein elevated the risk of in-hospital lethality by more than twice in both men and women (HR = 2.46; *p* < 0.001 and HR = 2.28; *p* < 0.001, respectively). *Conclusions*: The following differences between men and women with IS were determined: Acute in-hospital pulmonary complications, including COVID-19, significantly increased the risk of in-hospital lethality in the male group, but not in women. However, women suffering from DM had a significantly increased risk of in-hospital lethality compared with those women IS patients with AH or chronic ischemic heart disease (IHD). Increased C-reactive protein was associated with an elevated risk of in-hospital lethality both in male and female groups.

## 1. Introduction

Stroke remains an important public health challenge, impacting the world’s healthcare systems at large and emerging as the second most common single cause of death in Europe [1]. Ischemic stroke (IS), characterized by the sudden interruption of blood flow to the brain, represents a substantial portion of these cases. While the pathophysiology and risk factors for IS are well documented, an understudied aspect of this condition remains the sex-based discrepancies that might affect its management.

There is a need for the inclusion of sex as a biological variable in acute stroke research to improve the understanding of the underlying biological explanations for sex-based differences in patient outcomes, as well as to avoid misdiagnosis and undertreatment because of sex differences in presentation [2].

While stroke affects individuals of all ages, sexes, and backgrounds, mounting evidence suggests that sex could play some distinct role in shaping the incidence, outcomes, and treatment of this critical health issue.

Many of the findings for “sex differences” may represent the combined impact of both sex and gender, and gendered exposures may contribute to observed differences between men and women [3]. As women have a longer life expectancy, there is a higher stroke prevalence in women than in men [4,5], and this sex-based difference is expected to increase further over the next few decades [6,7]. Moreover, women tend to have higher mortality, especially in the oldest age groups [8]. At the onset of the disease, women usually are 4 to 5 years older, on average, than men and present with more comorbidities [9]. Both of these variables affect the severity of stroke and impact the timelines of acute stroke care.

Several studies performed before the COVID-19 pandemic reported greater stroke severity and worse outcomes in women [10], though the results from a large population-based prospective cohort study in the UK found no evidence of a worse outcome of stroke in women when adjusting for age and pre-morbid handicap [11]. Concern about nontraditional stroke symptoms in women presenting with stroke and the possibility of misdiagnosis, which can lead to inappropriate management, has been expressed [12].

In a pooled analysis of patients with acute stroke, survival at 3–6 months among women with IS was significantly higher than in men, though variations in out-of-hospital and in-hospital management could partly explain the disparities [13]. A recent systematic review identified amino acid metabolism as a potential mediator of different neurological outcomes in males and females with acute IS. Only a few articles have reported sex-specific subgroup analyses and a neurological outcome, indicating it is a key area for future research [2].

Some studies have showed that COVID-19 can increase the risk of stroke [14]. In the context of the COVID-19 pandemic, contradictory findings from previous studies addressing sex differences in cerebrovascular diseases demonstrate the need for further focused research [15,16]. A better understanding of the type and degree of inequities in stroke management and outcomes is important for improving health in IS patients. In Lithuania (which has a population of more than 2.6 million), there have been an estimated 1.3 million cases of COVID-19, with more than 9781 deaths until now (3.675 deaths per 1 M population) [17].

This study aims to evaluate the sex discrepancies in the clinical presentation of IS and its outcomes in patients admitted to Kaunas Hospital, Lithuanian University of Health Sciences (LUHS), Lithuania.

## 2. Materials and Methods

This is an observational analytical retrospective record-based single-center study. The study sample consists of 1809 patients admitted to Kaunas Hospital of the LUHS between 1 January 2020, and 27 February 2022, after a thorough evaluation at a tertiary stroke center in Kaunas. All the participants enrolled in the study—727 men and 1082 women—were suffering from acute IS and had absolute contraindications against interventional IS treatment: the study patients had either a time window of >4.5 h from the onset of symptoms, intracranial or spinal surgery in the previous three months, previous intracerebral bleeding, intracerebral neoplasm, infective endocarditis, aortic arch dissection, severe and uncontrolled AH, signs of present active internal bleeding, or some other absolute contraindication against systemic thrombolysis and/or thrombectomy.

Patient clinical examination, including instant brain computed tomography (CT) scan, assessment of the contraindications for interventional treatment or systemic thrombolysis, and the therapeutic decision, were performed at the tertiary stroke center. Further, these patients were transferred to the neurological department of Kaunas Hospital, LUHS, for conservative non-interventional IS management. At the tertiary stroke center, all the patients had been put on the standard treatment protocol for IS, without interventional treatment or thrombolysis, by the time they arrived to Kaunas Hospital of the LUHS. All study participants, except those with chronic or newly diagnosed atrial fibrillation, received antiplatelet therapy—100 mg of primarily acetylsalicylic acid daily as secondary stroke prophylaxis. Patients with atrial fibrillation were treated with subcutaneous low-molecular-weight heparin for anticoagulation during hospitalization, in accordance with standard non-interventional management protocols for IS. None of the study patients had antifibrotic therapy with nintedanib or any other antifibrotic agent, which can interact with direct oral anticoagulants and antiplatelets, potentially altering thromboembolic risk. Patients in our study did not receive the National Institutes of Health Stroke Scale (NIHSS) assessment of primary neurological deficit, as it is mandatory in Lithuania only before intravenous thrombolysis or mechanical thrombectomy.

The sociodemographic data; laboratory findings; comorbidities, including COVID-19; in-hospital complications; and outcome factors were obtained from patients’ medical records. “Sex” in our study refers to the biological characteristics of individuals; gender identity has not been evaluated separately.

Comorbidities were already pre-diagnosed before the admission to the neurological department.

The codes of the International Statistical Classification of Diseases and Related Health Problems, Tenth Revision, Australian Modification (ICD-10-AM), were denoted. Patients in our study cohort were comorbid with isolated AH (I11), DM (E11), complicated chronic IHD (I25) with heart failure (I50), atrial fibrillation and flutter (I48), or angina pectoris (I20). Other comorbidities included chronic tubulointerstitial nephritis (N11), electrolyte and acid–base balance disorders (E87), chronic kidney disease (N18), and Parkinson’s disease (G20). Acute in-hospital pulmonary (J18, J96) and kidney (N10, N17) complications were identified. The duration (in days) of the patient’s hospital stay until discharge or death was assessed.

### Statistical Analysis

Statistical analyses were carried out with IBM SPSS Statistics Version 27.0 (IBM Corp.; released 2020; IBM SPSS Statistics for Windows, Version 27.0; Armonk, NY, USA). Descriptive characteristics (prevalence rates, means, and standard deviations (SDs)) were calculated separately for men and women. The differences in means of variables between the sex groups were evaluated by deploying the *t*-test. A chi-squared test and z-test were used for assessing the differences in categorical variables. Laboratory findings were analyzed, stratifying to terciles of their absolute values. *p*-values < 0.05 were considered statistically significant. Hazard ratios (HRs) and 95% confidence intervals (CIs) were evaluated using the Cox proportional hazards regression for hospital lethality. Two models were assessed. Model 1 adjusted for age. Model 2 adjusted for age, COVID-19 status, specific comorbidities (AH, DM, and chronic IHD), and in-hospital complications.

## 3. Results

Table 1 shows the baseline characteristics of IS patients admitted to Kaunas Hospital of the LUHS differentiated by sex: 59.8% of the IS patients were women, and 40.2% were men. The mean age was significantly higher in women compared to men (*p* < 0.001). The prevalence of patients with IS plus specific comorbidities, such as AH, DM, and chronic IHD, was compared between the sex groups. In the group of men, the rate of IS patients with AH was significantly higher compared to women. The rate of IS patients with chronic IHD, though, was significantly higher in women compared to men.

The prevalence of COVID-19 was significantly higher in men compared to women (*p* < 0.05), while the proportions of the patients vaccinated for COVID-19 and the number of vaccinations in male and female groups did not differ significantly.

The rate of in-hospital kidney complications (N10, N18) was significantly higher in the group of women compared to men (*p* < 0.001). Also, the mean number of in-hospital complications was significantly higher in the group of women compared to men. Among the IS patients, the proportion of deceased was significantly higher in women compared to men (19.10% and 15.36%, respectively; *p* < 0.05). The distributions and means of laboratory findings also differed between the sex groups of IS patients. The means of creatinine and hematocrit were significantly higher in the group of men compared to women (*p* < 0.001).

The risk of in-hospital lethality for the specific comorbidities, in-hospital complications, COVID-19 status, and some laboratory findings according to sex are presented in Table 2. In men, after adjusting for age (Model 1), the risk of in-hospital lethality was 2.35 times higher in IS patients with chronic IHD compared to in IS patients with AH. The risk of in-hospital lethality in men suffering from COVID-19 was 2.94 times higher compared to men without the disease. After the evaluation of the prognostic value of in-hospital complications in the group of men, we determined that the risk of in-hospital lethality was significantly higher in patients with pulmonary complications (J18, J96) and kidney diseases (N10, N17) compared with men without these complications (the risk was higher by almost five and three times, respectively). The laboratory findings, such as increased creatinine and C-reactive protein, revealed an elevated risk of in-hospital lethality in men (by 1.49 and 2.88 times, respectively). After an additional adjustment for all variables included in the Cox regression model (Model 2), only a COVID-19 diagnosis (HR = 1.53; *p* = 0.02), in-hospital pulmonary complications (HR = 1.91; *p* = 0.008), and increased C-reactive protein (HR = 2.46; *p* < 0.001) significantly elevated the risk of in-hospital lethality in men.

In women, after adjusting for age (Model 1), the risk of in-hospital lethality was about two times higher in IS patients with DM and with chronic IHD compared to those with isolated AH. After the evaluation of the prognostic value of in-hospital complications in the group of women, we found that the risk of in-hospital lethality was significantly higher in women with pulmonary and kidney complications compared with women without these complications. The laboratory findings, such as increased creatinine, C-reactive protein, and hematocrit, showed a significantly elevated risk of in-hospital lethality in women (1.32, 2.66, and 1.26 times, respectively). After an additional adjustment for all variables included in the Cox regression model (Model 2), the risk of in-hospital lethality was significantly higher in women with comorbid DM compared to in those with comorbid isolated AH (HR = 2.25, *p* = 0.007). Further, in the group of women, the increased C-reactive protein elevated the risk of in-hospital lethality by more than two times (HR = 2.28; *p* < 0.001).

## 4. Discussion

The findings of this study give some insights into the presentations of acute IS, specifically examining the impact of sex, comorbidities, and in-hospital complications during the time of the COVID-19 pandemic. This study aligns with existing literature suggesting few sex-specific differences in patients with IS. Notably, women presented with a higher mean age compared to men, consistent with a global trend according to which women tend to be older at the onset of stroke [18,19,20,21,22,23,24]. This age disparity contributes significantly to the differences in comorbidity profiles and stroke outcomes. Hence, women have been shown to exhibit a significantly higher prevalence of chronic IHD [25], aligning with similar sex-specific patterns in cardiovascular comorbidities already reported for a Spanish cohort of cardioembolic stroke patients, where atrial fibrillation and valvular disease were more prevalent among women and IHD predominated in men [26]. However, in our study, atrial fibrillation was not analyzed as a separate variable, limiting direct comparison with cardioembolic stroke etiology in other populations and restricting the availability of data on the use of anticoagulation therapy, including low-molecular-weight heparins, direct oral anticoagulants, and vitamin K antagonists. Future registry updates are meant to include these data to support more refined etiological stroke classification and improved evaluation of treatment strategies.

Females have been shown to bear a disproportionate burden of stroke mortality and disability. Biological sex and sociocultural gender contribute to differences in stroke risk factors, clinical assessment, treatment, and outcomes. Significant differences have been found in the strength of associations between stroke risk factors and risk factors specific to females. Older age, hypertension, and smoking appear to be stronger drivers of cardiovascular disease in women, whereas lipid metrics appear to be stronger risk determinants for men. These findings highlight the importance of sex-specific preventive strategies and suggest priority targets for intervention in men and women [21]. In addition, there are differences in stroke incidence rates, response to treatment, and stroke outcomes in women [3].

Sex and gender, as biological and social factors, have a significant impact on health outcomes. Among biological factors, sex differences in vascular physiology may be one of the specific mechanisms contributing to observed differences in clinical features, response to treatment, and clinical outcomes in several vascular disorders. The literature has focused primarily on comparing cerebral vascular hemodynamics between women and men [27]. Decreased middle cerebral artery blood flow velocity and blood flow pulsatility are factors that determine the pathogenesis of age-related cerebrovascular diseases. It is not known whether middle cerebral artery blood flow velocity and the rate of change in blood flow pulsatility support the hypothesis of gender-specific trajectories with aging. It has been found that lower middle cerebral artery blood flow velocity and the rate of increase in blood flow pulsatility are significantly greater in women compared with men. This could partly explain the differences in morbidity and mortality from cerebrovascular diseases between the sexes [28].

Menopause and its associated hormonal changes (decrease in estrogen) are associated with chronic diseases such as cardiovascular and metabolic diseases, which may be difficult to distinguish from the effects of ageing. In addition, postmenopausal women are at increased risk of cerebrovascular disease, which is associated with decreased cerebral blood flow and cerebrovascular reactivity, but the direct impact of menopause on cerebrovascular function is unclear [29].

Women have a higher lifetime stroke rate than men, largely due to a sharp increase in stroke risk in older women after menopause. Women also have a higher prevalence of stroke risk factors, including hypertension and atrial fibrillation in the postmenopausal period, and middle-aged women have a higher prevalence of abdominal obesity and metabolic syndrome compared with men [30]. The risks of oral contraceptives, hormone therapy, and surgery for carotid artery stenosis in women are still debated [31].

Treatment differences with anticoagulants and rhythm control in atrial fibrillation are also different for women and men [20,32]. According to various studies, women are less likely to receive anticoagulant treatment and treatment to maintain heart rhythm than men [33].

It is well established that sex is an important modifier of physiology and disease, and sex differences in disease prevalence, manifestation, and response to treatment are rooted in the genetic differences between men and women [33,34].

In our study, both men and women had a higher risk of in-hospital lethality in IS patients with chronic IHD compared to IS patients with AH, aligning with findings reported from Romania [35], where the presence of IHD was also found to be an important predictor of in-hospital mortality.

The significance of DM for the risk and the presentation of cardio- and cerebrovascular diseases, including IS, is well documented already [36]. An interaction between sex and diabetes mellitus for in-hospital lethality, however, remains somewhat equivocal. Previous studies reported that coexisting DM correlated with a significantly higher lethality in male, but not in female, acute myocardial infarction patients [36,37], while our study presents contradictory results when analyzing the in-hospital lethality of IS patients. This finding aligns with previous stroke-specific research by Arboix et al., who demonstrated that female sex had a negative impact on prognosis in type 2 diabetic patients with IS, with women showing worse functional outcomes and higher mortality rates compared to diabetic men with stroke [38]. These sex-specific differences in diabetic stroke patients may reflect distinct pathophysiological mechanisms or varying responses to metabolic stress between men and women during acute cerebrovascular events and might support the need for sex-specific approaches in stroke care.

As applied in our study, biological sex, defined by biological characteristics of individuals including genetic, biologic, and physiological expression, could be compared with results from other studies admitting that there are important differences between males and females in terms of symptoms, the course of disease, and therapeutic response due to molecular, genetic, and epigenetic mechanisms related to biological sex. However, there is contradictory information about gender inequity and acute stroke treatment [15,39,40].

Ralph Weber, in a nationwide analysis of all hospitalized IS patients in Germany, found that men of all age groups had a significantly higher probability of receiving stroke unit treatment and lower in-hospital mortality; no disparity was observed in the use of intravenous thrombolysis, while women of all ages were treated more often with mechanical thrombectomy [41]. In a recent single-center study analyzing sex-specific differences in stroke patients between 2014 and 2020, worse functional outcome after acute IS in women was mainly driven by older age, poorer social independence, higher stroke severity, and more stroke due to large vessel occlusion in women, but that worse outcome was not independently sex-related. Women presented less frequently when more severe neurological deficit occurred [40].

In accordance with earlier findings indicating that women continue to face a greater lifetime morbidity and mortality from stroke [2,42], our data indicates that women constituted both the majority of the acute IS sample and the majority of the deceased inpatients during the study time.

In the study cohort, in-hospital deaths among IS patients were attributable to both neurological and non-neurological conditions. Neurological causes, including massive cerebral infarction, cerebral edema, hemorrhagic transformation, and recurrent ischemic events, predominated as the primary cause of death, particularly among patients with more severe stroke presentations. Non-neurological conditions, such as aspiration pneumonia, pulmonary embolism, and acute renal failure, were also prevalent, especially in older patients and those with multiple comorbidities. When stratified by sex, women more frequently died from primary neurological causes, possibly reflecting a higher proportion of large infarcts and older age at onset.

The reported findings align with some previous international evidence on sex-specific in-hospital mortality following IS. An Italian systematic review found that women had 34% increased odds of in-hospital death (OR 1.34; 95% CI 1.04–1.74) [43]. Similarly, a prospective cohort of 296 stroke patients showed a trend toward higher acute-phase mortality in women, possibly related to a lower proportion of lacunar strokes and reduced aspirin use [44]. In a Spanish cohort of 13,932 stroke patients, crude in-hospital mortality was higher in women, but again, this difference was no longer significant after adjusting for stroke severity and age. In this study, the leading cause of death in women was the stroke itself [45].

It is important to consider the impact of the COVID-19 pandemic, which has led to less frequent or delayed access to healthcare facilities as well as an excess mortality rate in Lithuania (2.8 deaths per 1000 population (2020–2021)) [46]. A rural, multi-center retrospective cohort study of hospitalized patients diagnosed with COVID-19 performed in 2020 found that at ages greater than 65 years, congestive heart failure, CKD, acute respiratory failure, cancer, and intensive care unit stay were associated with a higher risk of in-hospital death [47]. The short-term and long-term impact of COVID-19 on cardiovascular health needs further investigation.

Delving into laboratory findings—namely, creatinine, C-reactive protein, and hematocrit—has revealed several sex-specific variations. Men showed higher mean levels of creatinine and hematocrit, which can be addressed as an additional risk factor for cardiovascular disease [48]. Women displayed higher C-reactive protein levels, and for both men and women, increased C-reactive protein was associated with an elevated risk of in-hospital lethality. D-dimers, another important and well-established prognostic biomarker in various thrombotic and inflammatory conditions, including ischemic stroke and COVID-19, were available only in exceptional cases in our study and were therefore excluded from further analysis. This represents a limitation, as it restricts the ability to perform a more comprehensive evaluation of thromboinflammatory risk and prognosis in stroke patients.

As the study took place during the outbreak of the coronavirus disease (COVID-19), during which significantly higher rates of severe clinical type of COVID-19 were observed in older patients compared to younger ones [49], we assumed the possibility of its impact on elderly populations. 

It is already known that COVID-19 has an impact on the presentation of IS with several pathophysiological mechanisms, with hypercoagulability, general inflammation, and vasculopathy being of considerable importance [50]. Our study explored the impact of COVID-19 on IS outcomes. In our study, men suffering from IS demonstrated a higher prevalence of COVID-19 along with a higher COVID-19-related risk of in-hospital lethality, which is consistent with existing data suggesting that men may be more susceptible to severe outcomes of COVID-19 [51]. Pulmonary inpatient complications were significantly related to the risk of hospital lethality for IS in men, which could be also related to COVID-19. Nevertheless, comorbidity with COVID-19 did not significantly increase the risk of hospital lethality in women, indicating potential sex-specific differences in the interaction between the pathophysiology of COVID-19 and IS. IS in patients with COVID-19 was also found to be associated with endotheliopathy and a systemic inflammatory response in patients with vascular risk factors [52]. Further studies are needed to clarify the mechanisms of IS in COVID-19 patients with risk factors and comorbidities and enhance their therapy.

The analysis of in-hospital complications revealed that women exhibited a higher rate of urinary tract complications, corresponding to an anatomically different female urogenital system [53]. The overall number of in-hospital complications, again, was significantly higher in women compared to men. Further, the in-hospital lethality was significantly higher in women compared to men, as well. This suggests that women may be more vulnerable to certain in-hospital complications, contributing to their increased risk of in-hospital lethality [54].

In further studies, we will strive to prioritize the development of a comprehensive stroke database that incorporates structured clinical data to facilitate more precise etiological classification of cerebrovascular events and enhance our understanding of sex-specific variations in stroke pathophysiology and therapeutic response. Our research will focus on integrating artificial intelligence technologies, including machine learning algorithms and natural language processing systems, to advance analytical capabilities in stroke medicine.

## 5. Limitations

An evaluation of the study’s limitations is warranted. The study sample consisted of IS patients who were evaluated at a tertiary-level stroke center. All the patients evaluated were non-elective for intravenous thrombolysis or mechanical thrombectomy. This could be indicative of either a worse clinical situation at presentation or a longer time interval from the onset of symptoms. Patients in our study did not receive the National Institutes of Health Stroke Scale (NIHSS) assessment of primary neurological deficit, as it is mandatory in Lithuania only before intravenous thrombolysis or mechanical thrombectomy. All patients in our study cohort had absolute contraindications to these interventional treatments. The absence of a baseline neurological deficit assessment represents a significant limitation, as stroke severity is highly important for ischemic stroke outcomes and prognosis comparison between sexes. This limitation should be acknowledged as it hinders precise risk stratification and the meaningful comparison of outcomes across different patient subgroups.

A notable limitation of our study is the absence of the classification of ischemic stroke subtypes according to TOAST [55]. In this study, we did not routinely differentiate cases into large-artery atherosclerosis, cardioembolic, small-vessel occlusion, or other determined and undetermined causes. This unfortunately restricts deeper insight into risk profiles and recurrence likelihood. Additionally, information on the topographic distribution of cerebral infarcts—anterior versus posterior circulation or specific vascular territories (middle cerebral artery, brainstem, etc.)—was not systematically captured in the context of our study. This limits the ability to assess anatomical patterns of stroke and their association with in-hospital outcomes. Future registry updates and prospective studies should aim to include standardized imaging-based data on both etiological subtypes and lesion topography, as these variables are critical for clinical stratification and targeted stroke prevention strategies.

Furthermore, for now, we lack a longer-term follow-up, and it is still difficult to evaluate the effect of the sex-specific factors, comorbidities, and complications on post-hospital lethality. This gap is meant to be filled by our upcoming study. Differences in lifestyle, treatment, and early diagnostic aspects that have not been evaluated in this study sample might also have an additional value and explain more variables attributed to sex in IS among men and women.

## 6. Conclusions

This study highlights the significance of sex-specific factors, pre-existing conditions, and in-hospital complications in predicting the risk of in-hospital lethality among IS survivors. The following differences between men and women with IS were determined: Acute in-hospital pulmonary complications, including COVID-19, significantly increased the risk of in-hospital lethality in the male group, but not in women. However, women suffering from DM had a significantly increased risk of in-hospital lethality compared with those women IS patients with AH or IHD. Increased C-reactive protein was associated with an elevated risk of in-hospital lethality both in male and female groups. This study emphasizes the importance of addressing the varied factors in men and women that contribute to in-hospital lethality in IS patients.

## Figures and Tables

**Table 1:** Baseline characteristics of ischemic stroke patients admitted to Kaunas Hospital of the Lithuanian University of Health Sciences.

Variables	Male	Female	*p*
Sex, *n* (%)	727 (40.2)	1082 (59.8)	
Age in years, mean (SD)	72.3 (11.0)	79.7 (9.6)	**<0.001**
Age group, years (%)			
25–64	26.0	7.6	**<0.001**
65–84	61.8	61.2	0.795
85+	12.2	31.2	**<0.001**
Patients with ischemic stroke (IS) + specific comorbidities, *n* (%)			
AH	323 (44.4)	341 (31.5)	**<0.001**
DM	85 (11.7)	129 (11.9)	0.897
Chronic IHD	319 (43.9)	612 (56.6)	**<0.001**
COVID-19 (U07), *n* (%)	80 (11.0)	86 (7.9)	**0.027**
Number of comorbidities, mean (SD)	1.6 (0.9)	1.8 (1.0)	**<0.001**
In-hospital complications, *n* (%)			
** Pulmonary complications**			
J18, J96	72 (9.9)	100 (9.2)	0.638
** ** **Kidney complications**			
N10, N17	40 (5.5)	108 (10.0)	**<0.001**
Number of in-hospital complications, mean (SD)	0.2 (0.5)	0.3 (0.6)	**0.047**
Number of patients with at least one in-hospital complication, *n* (%)	121 (16.6)	217 (20.1)	0.068
Length of hospitalization, mean (SD)	14.8 (11.9)	14.7 (11.9)	0.466
Vaccination for COVID-19, *n* (%)	76 (10.5)	87 (8.0)	0.079
Number of vaccinations, *n* (%)			
1	9 (11.8)	8 (9.2)	0.581
2 and more	67 (88.2)	79 (90.8)	
Deceased, *n* (%)	111 (15.3)	207 (19.1)	**0.034**
Time to death, days, mean (SD)	10.5 (11.7)	9.1 (9.0)	0.148
**Laboratory findings**			
Creatinine µmol/L, mean (SD)	93.1 (61.4)	83.0 (51.2)	**<0.001**
**Creatinine tercile intervals**			
I tercile	28.1–71.4	18.0–62.1	
II tercile	71.5–100.0	62.2–83.8	**<0.001**
III tercile	100.1–802.9	83.9–681.6	
C-reactive protein mg/L, mean (SD)	37.8 (57.4)	34.0 (47.3)	0.126
**C-reactive protein tercile intervals**			
I tercile	0.1–6.7	0.1–8.3	
II tercile	6.8–34.2	8.4–31.4	0.334
III tercile	34.3–405.5	31.5–385.6	
Haematocrit %, mean (SD)	41.4 (6.0)	38.9 (5.4)	**<0.001**
**Haematocrit tercile intervals**			
I tercile	20.4–39.3	20.8–36.8	
II tercile	39.4–44.1	36.9–41.1	**<0.001**
III tercile	44.2–64.5	41.2–57.6	

**Table 2:** Risk of hospital lethality for ischemic stroke in combination with comorbidities, in-hospital complications, COVID-19 status, and laboratory findings according to sex.

		MALE	*n* = 727			FEMALE	*n* = 1082	
Variables	Model 1		Model 2		Model 1		Model 2	
	HR	95% CI	HR	95% CI	HR	95% CI	HR	95% CI
**Age**			1.02	0.99–1.04			**1.02**	1.00–1.04
**IS + comorbidities**								
AH	1		1		1		1	
DM	1.31	0.65–2.66	1.30	0.56–3.02	**2.28**	1.38–3.74	**2.25**	1.25–4.05
Chronic IHD	**2.35**	1.47–3.74	1.68	0.94–2.98	**1.90**	1.32–2.73	1.24	0.80–1.91
**COVID-19 status** **(Yes vs. No)**	**2.94**	1.73–5.02	**1.53**	1.07–2.20	1.20	0.71–2.03	0.95	0.67–1.36
**In-hospital complications** **(Yes vs. No)**								
Pulmonary complications (J18, J96)	**4.89**	3.30–7.25	**1.91**	1.19–3.08	**2.89**	2.09–3.99	1.26	0.84–1.88
Kidney complications (N10, N17)	**2.94**	1.75–4.93	0.75	0.39–1.42	**2.30**	1.64–3.22	1.09	0.72–1.64
**Laboratory findings,** per one tercile								
Creatinine	**1.49**	1.13–1.95	1.27	0.94–1.71	**1.32**	1.08–1.61	1.23	0.99–1.53
C-reactive protein	**2.88**	2.08–3.99	**2.46**	1.70–3.55	**2.66**	2.10–3.35	**2.28**	1.74–2.98
Haematocrit	1.16	0.89–1.50	1.25	0.96–1.64	**1.26**	1.03–1.53	1.22	0.99–1.49

## Data Availability

All relevant data are within the paper.
